# Functional Limitation and Favorable Mental-Health Self-Appraisal Among U.S. Adults Aged 50 Years or Older with Multimorbidity: A Behavioral-Science Analysis of the 2023 Medical Expenditure Panel Survey

**DOI:** 10.3390/bs16060841

**Published:** 2026-05-22

**Authors:** Minyang Zhang, Juan Du, Yidan Ding, Yichen Xiao, Yumei Jiang, Jie Liu

**Affiliations:** 1 School of Journalism and Information Communication, Huazhong University of Science and Technology, Wuhan 430074, China; 2 College of Literature, Nanjing University, Nanjing 210019, China; 3 School of Physical Education, Huazhong University of Science and Technology, Wuhan 430074, China

**Keywords:** multimorbidity, functional limitation, mental-health self-appraisal, older adults, health psychology, behavioral adaptation, MEPS, survey-weighted analysis, positive mental health

## Abstract

How older adults psychologically appraise their health while managing multiple chronic conditions is a behavioral-science question as much as a clinical one. This study estimated the weighted prevalence of favorable mental-health self-appraisal, identified its behavioral, social, and functional correlates, and compared the relative salience of diagnosed-condition burden and functional limitation among U.S. adults aged ≥ 50 years with multimorbidity. This retrospective cross-sectional secondary analysis used the 2023 Medical Expenditure Panel Survey (MEPS) Full Year Consolidated Data File (HC-251). Multimorbidity was defined as at least two diagnosed chronic priority conditions. The primary outcome represents favorable mental-health self-appraisal, derived from MNHLTH53 (excellent/very good/good vs. fair/poor). Covariates were organized using Andersen’s Behavioral Model and health-psychology concepts of adaptation, resources, and lived functional burden. Weighted prevalence estimates and survey-weighted logistic regression models were fitted using PERWT23F, VARSTR, and VARPSU. Robustness checks examined a stricter outcome threshold, proxy adjustment/non-proxy restriction, and a physical-health extension model. The analytic sample included 5523 respondents, representing approximately 77.9 million U.S. adults aged ≥ 50 years with multimorbidity. The weighted prevalence of favorable perceived mental-health self-appraisal was 86.6% (95% CI 85.4–87.7). In the fully adjusted core model (complete-case *n* = 5330), age 65–74 years (aOR 1.52, 95% CI 1.17–1.98) and age ≥ 75 years (aOR 1.79, 95% CI 1.36–2.36) were associated with higher odds of favorable appraisal. Lower odds were observed for Hispanic respondents, non-Hispanic Asian respondents, lower educational attainment, lower income, non-employment, ≥4 diagnosed conditions, and any functional limitation. The strongest inverse association was limitation status (aOR 0.32, 95% CI 0.27–0.39). Sensitivity analyses were directionally consistent. Favorable mental-health self-appraisal remained common in this medically complex older population, but it was socially and functionally patterned. Functional limitation appeared more behaviorally salient than diagnosis count alone. Because the analysis was cross-sectional and based on household-interview reported measures, these results should be interpreted as associations rather than causal effects.

## 1. Introduction

Mental health is a core component of health, functioning, and social participation rather than merely the absence of psychiatric disorder. The World Health Organization defines mental health as a state of well-being that enables people to cope with the stresses of life, realize their abilities, work productively, and contribute to their communities (World Health Organization, 2025). For behavioral science, this definition is important because it places appraisal, coping, role participation, and everyday functioning at the center of mental health. In later life, these processes are shaped not only by symptoms or diagnoses but also by functional capacity, social roles, economic resources, health-care access, and adaptation to chronic illness.

Multimorbidity, commonly defined as the coexistence of two or more chronic conditions, has become a central challenge of aging populations (Barnett et al., 2012; Salive, 2013; Carlson & Yarns, 2023; Skou et al., 2022). It is associated with treatment burden, polypharmacy, fragmented care, lower quality of life, and functional decline (Salive, 2013; Skou et al., 2022; Ryan et al., 2015; Makovski et al., 2019). However, multimorbidity is not a single behavioral experience. Diagnosis counts, severity-weighted indices, symptoms, and functional restrictions capture different aspects of burden (Huntley et al., 2012; Stirland et al., 2020). From a health-psychology standpoint, the distinction between the number of conditions a person has and the extent to which those conditions constrain daily life is especially important.

The dominant literature on multimorbidity and mental health remains deficit-oriented. Systematic reviews and meta-analyses consistently link multimorbidity with depression, anxiety, cognitive decline, and poorer psychosocial outcomes (Palmese et al., 2025; Pundhir et al., 2025; Du et al., 2025). These studies are essential, but they leave a complementary behavioral-science question underdeveloped: which older adults continue to appraise their mental health favorably despite multiple chronic conditions? A strengths-based outcome is not simply the inverse of distress. It can reveal adaptation, perceived control, preserved participation, and resource availability among people who remain medically complex.

This study was informed by behavioral and health-psychology perspectives that treat perceived mental health as an appraisal shaped by adaptation, resources, and participation. Dual-continua and response-shift perspectives suggest that favorable appraisal may persist despite chronic illness (Keyes, 2002, 2005; Stephens et al., 2023; Lamers et al., 2011; Wang et al., 2025), whereas functional limitation may make illness burden more psychologically salient by constraining autonomy and valued activities (Sprangers & Schwartz, 1999; Whitmore et al., 2022; van Loon et al., 2023). These perspectives support distinguishing diagnosis count from lived functional burden when examining mental-health appraisal in later-life multimorbidity (Carstensen et al., 1999; Charles, 2010; Cosco et al., 2017; Ding et al., 2025).

Social context is also central. Multimorbidity is socially patterned, and its lived burden is shaped by education, income, employment, household resources, and neighborhood disadvantage (Afshar et al., 2015; Moreno-Juste et al., 2023; Domènech-Abella et al., 2018; Ingram et al., 2021). Mental health is similarly shaped by social determinants across the life course (Allen et al., 2014; Luo et al., 2025). Among older adults, lower socioeconomic position may reduce access to coping resources, social participation, preventive care, and environmental support. Accordingly, favorable mental-health self-appraisal in multimorbidity should not be interpreted as an individual trait alone; it is likely patterned by enabling resources and structural vulnerability.

Measurement requires caution. A global perceived mental-health item is not equivalent to a diagnostic interview or a multi-item well-being scale, but it is useful in population surveys because it captures a broad evaluative judgment about one’s mental or emotional health (Ahmad et al., 2014; Fung et al., 2024; Hays et al., 2017; McAlpine et al., 2018). Reviews of self-rated mental health show meaningful associations with distress, service use, quality of life, and social determinants, although responses may vary across cultural and reporting contexts (Ahmad et al., 2014; McAlpine et al., 2018). In this manuscript, the outcome is therefore described as favorable mental-health self-appraisal rather than as a clinical diagnosis of positive mental health. This wording better matches the construct measured by the Medical Expenditure Panel Survey (MEPS). Prior MEPS-based studies in behavioral sciences have examined perceived mental health or psychological well-being in pain-related and arthritis populations (Axon & Chien, 2021; Awad & Axon, 2022; Axon & Kim, 2023; Axon & Agu, 2024).

These studies demonstrate the value of MEPS for behavioral and health-psychology research, but they do not fully answer the present question for three reasons. First, they were largely condition-specific rather than focused on a broader older-adult multimorbidity population. Second, they did not place diagnosis count and functional limitation in direct comparison as alternative indicators of illness burden. Third, they did not foreground favorable mental-health self-appraisal as an outcome shaped jointly by social resources, functional participation, and chronic-disease burden. This leaves a specific gap: among older adults who already meet a multimorbidity definition, it remains unclear whether favorable mental-health self-appraisal is more strongly patterned by the number of diagnosed conditions or by the extent to which illness constrains daily life.

The present study examined U.S. adults aged ≥ 50 years with multimorbidity using the 2023 MEPS Full Year Consolidated Data File (HC-251). Covariates were organized with Andersen’s Behavioral Model as an analytic heuristic (Andersen, 1995; Awad & Axon, 2022), while interpretation was grounded in health psychology, behavioral adaptation, and participation under chronic illness burden. The study addressed three questions: (1) What proportion of older adults with multimorbidity report favorable mental-health self-appraisal? (2) Which predisposing, enabling, and need/functioning characteristics are independently associated with that favorable appraisal? (3) Are the associations robust at a stricter outcome threshold and in sensitivity analyses addressing proxy reporting and perceived physical health? We expected functional limitation to show a stronger inverse association with favorable appraisal than diagnosis count alone, because limitation more directly captures the lived behavioral consequences of illness.

## 2. Materials and Methods

### 2.1. Study Design and Data Source

This study was a retrospective cross-sectional secondary analysis of the 2023 Medical Expenditure Panel Survey (MEPS) Full Year Consolidated Data File (HC-251). According to the Agency for Healthcare Research and Quality (AHRQ), HC-251 was released in August 2025 and contains nationally representative person-level data for the U.S. civilian noninstitutionalized population for the calendar year 2023 when the full-year person weight is applied (Agency for Healthcare Research and Quality, 2025b). The file includes 18,919 persons overall and 18,463 persons with a positive person-level weight (Agency for Healthcare Research and Quality, 2025a, 2025b). HC-251 is suitable for this behavioral-science analysis because it combines chronic condition indicators, functioning, employment, family income, insurance, usual source of care, perceived health, and survey-design variables in a single national household dataset.

MEPS household-component data are collected through household interviews in which a designated reporting-unit respondent may provide information for other household members. AHRQ documentation also distinguishes reporting-unit members from non-reporting-unit proxy respondents and identifies round-specific proxy variables, including PROXY53. For this reason, the primary outcome is interpreted as an interview-based perceived mental-health appraisal rather than a purely self-administered psychological scale. Proxy-related sensitivity analyses were included to assess whether the core pattern depended on reporting context.

### 2.2. Study Population and Sample Selection

The analytic population was restricted to adults aged ≥ 50 years with a positive full-year person-level weight (PERWT23F > 0). The age cutoff of ≥50 years was chosen for conceptual and analytic reasons. Conceptually, age 50 marks the transition from midlife into later-life chronic-disease accumulation and allows comparison of adults approaching older age with those aged 65–74 years and ≥75 years. Analytically, this cutoff retained a sufficiently large under-65 comparison group while focusing the study on an age range in which multimorbidity and functional limitation are increasingly relevant. The cutoff was specified before outcome modeling and was not selected based on observed mental-health self-appraisal patterns. PERWT23F is the HC-251 full-year person weight used to produce person-level estimates for the U.S. civilian noninstitutionalized population in the calendar year 2023. In this study, unweighted counts describe the number of HC-251 records retained at each sample-selection step, whereas weighted totals describe the corresponding population estimates obtained by summing PERWT23F among respondents meeting the analytic criteria. Respondents were then required to have complete information for the diagnosis-based multimorbidity definition and a valid Round 5/3 perceived mental health status value (MNHLTH53 coded 1–5). Of the 18,919 records in HC-251, 18,463 had a positive person-level weight, 8205 were aged ≥ 50 years, 8175 had complete data for the diagnosed-condition count, 5574 met the primary multimorbidity definition, and 5523 had valid MNHLTH53 values and formed the final analytic sample. After applying PERWT23F, these 5523 unweighted respondents represented approximately 77.9 million U.S. civilian noninstitutionalized adults aged ≥ 50 years with multimorbidity in 2023. Thus, subsequent weighted percentages and prevalence estimates should be interpreted as estimates for this target population rather than as simple proportions of the unweighted analytic sample.

### 2.3. Outcome Variable

The primary binary outcome classified excellent, very good, and good responses as favorable mental-health self-appraisal and contrasted them with fair or poor (Agency for Healthcare Research and Quality, 2025a). This cutoff point was chosen for both conceptual and methodological reasons. Conceptually, the study aimed to identify respondents reporting at least a favorable global appraisal of their mental health rather than to diagnose positive mental health or psychiatric disorder. Methodologically, grouping excellent, very good, and good responses together maintained comparability with prior MEPS studies of perceived mental or psychological health (Axon & Chien, 2021; Awad & Axon, 2022; Axon & Kim, 2023; Axon & Agu, 2024; Zhai et al., 2026), while the term appraisal avoids overinterpreting a single global item as a diagnostic or multidimensional well-being scale. Because the boundary between “good” and “fair” may be interpreted differently across individuals and social groups, a stricter sensitivity analysis redefined the favorable outcome as excellent or very good versus good, fair, or poor.

### 2.4. Primary Multimorbidity Definition

The primary multimorbidity definition was intentionally conservative and diagnosis-based. Respondents were classified as having multimorbidity when at least two of the following chronic diagnosed condition domains were present in HC-251: high blood pressure (HIBPDX), composite heart disease (coronary heart disease, angina, myocardial infarction, or other heart disease), stroke (STRKDX), emphysema (EMPHDX), high cholesterol (CHOLDX), cancer (CANCERDX), arthritis (ARTHDX), diabetes (DIABDX_M18), and asthma (ASTHDX) (Agency for Healthcare Research and Quality, 2025a). A composite heart-disease indicator was used to avoid double-counting conceptually overlapping cardiovascular diagnoses.

Several HC-251 priority-condition indicators were not included in the primary multimorbidity count. This decision was made to keep the exposure construct conservative, diagnosis-based, and comparable across respondents rather than to imply that the excluded conditions are clinically unimportant. Joint pain was excluded because it is primarily symptom-based and may reflect multiple underlying musculoskeletal or inflammatory conditions rather than a distinct diagnosed chronic-condition domain. Chronic bronchitis was excluded because the consolidated-file item may be more episodic or respiratory-symptom-oriented than the stable diagnosed domains retained in the primary count. COVID-19 and long-COVID indicators were excluded because their timing, chronicity, severity, and etiologic heterogeneity differ from established chronic priority conditions and could have introduced ambiguity into an older-adult multimorbidity construct based on longstanding diagnosed conditions. These exclusions were specified before final modeling to reduce construct heterogeneity and improve interpretability. Nevertheless, the resulting count may not be directly comparable with broader multimorbidity definitions that include symptom-based, respiratory-episode, post-viral, or conditions-file-based indicators; this comparability issue is acknowledged in the limitations.

### 2.5. Covariates and Conceptual Organization

Covariates were selected a priori and organized using Andersen’s Behavioral Model as an analytic organizing device (Andersen, 1995; Awad & Axon, 2022). Predisposing factors were variables that characterize demographic position, social identity, or life-stage context before immediate illness burden was considered: age group (50–64, 65–74, and ≥75 years), sex, race/ethnicity, marital status, educational attainment, and census region. Education was placed in the predisposing block because it reflects relatively stable human capital accumulated earlier in the life course, although it also has socioeconomic implications. Enabling factors were variables representing current material, institutional, or access-related resources that may shape coping opportunities and care access: family poverty category, employment status, insurance coverage, and usual source of care. Employment was treated as enabling because it indexes current role participation, financial security, and access to work-related resources, rather than being interpreted only as a demographic attribute. Usual source of care was treated as enabling because it reflects care continuity and access infrastructure rather than illness severity. Need/functioning factors represented current illness burden and daily-life functional consequences: diagnosed-condition burden and any functional limitation. This organization was prespecified before final model estimation; descriptive distributions were reviewed only to verify coding feasibility and avoid sparse categories, not to select covariates based on outcome associations. Table 1 maps each analytic construct to its corresponding MEPS variable(s) and coding decisions.

**Figure 1:**
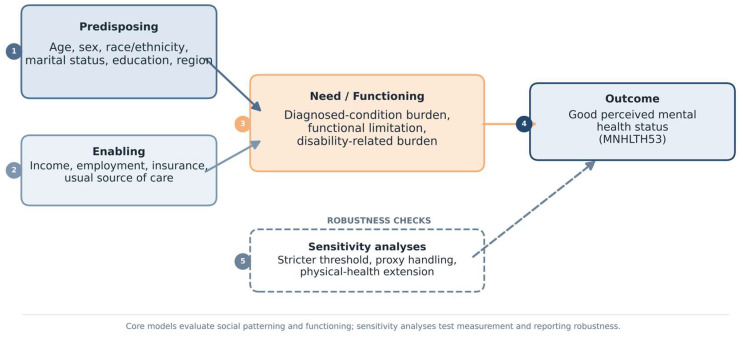
Conceptual model for favorable mental-health self-appraisal in later-life multimorbidity. Blue boxes indicate predisposing, enabling, and outcome domains; the orange box indicates the need/functioning domain; and the dashed box indicates robustness checks and sensitivity analyses. Solid arrows represent the core analytic pathways, and the dashed arrow represents sensitivity and extension analyses.

Perceived physical health status (RTHLTH53) was not included in the primary core model because of the strong possibility of shared-method variance with perceived mental health status. In MEPS, both are global evaluative ratings collected in the same household interview context (Agency for Healthcare Research and Quality, 2025a). Including perceived physical health in the primary model could collapse distinct behavioral and functional pathways into a common evaluative tendency. Instead, perceived physical health was introduced only in a planned extension model. Proxy-reporting status at Round 5/3 (PROXY53) was also treated as a robustness variable rather than a core explanatory construct. Figure 1 summarizes the analytic organization and planned robustness checks.

### 2.6. Statistical Analysis

All analyses accounted for the complex survey design of MEPS. In accordance with HC-251 documentation, person-level analyses that did not use Self-Administered Questionnaire variables applied PERWT23F as the full-year person weight and used VARSTR and VARPSU for Taylor-series variance estimation (Agency for Healthcare Research and Quality, 2025a, 2025b). Weighted descriptive statistics were used to summarize the analytic sample and estimate the prevalence of favorable mental-health self-appraisal overall and across selected strata. Survey-adjusted bivariate contrasts were examined descriptively, and multivariable associations were estimated with survey-weighted logistic regression.

A nested modeling strategy was used to make the build-up of adjustment transparent. Model 1 included predisposing factors only. Model 2 added enabling factors. Model 3 added need/functioning factors and served as the primary core model. Adjusted odds ratios (aORs) with 95% confidence intervals (CIs) are reported in the main text and tables. To improve interpretability beyond odds ratios, adjusted predicted probabilities were calculated from the primary core model for combinations of diagnosed-condition burden and limitation status. Because the study design was cross-sectional, all estimates were interpreted as associations rather than determinants or effects. In addition, because perceived mental health, chronic-condition indicators, and limitation status were based on household-interview reports, estimates were interpreted as associations between reported characteristics rather than clinically verified causal pathways. All tests were two-sided, and *p* < 0.05 was considered statistically significant.

Additional diagnostic checks were conducted for the fully adjusted complete-case core model to evaluate specification stability. Weighted variance inflation diagnostics from the model design matrix showed no evidence of problematic collinearity; the largest individual variance inflation factor was 2.03, and the largest values for socioeconomic, access, burden, and functioning variables were all below this level. Calibration was assessed by comparing weighted observed and predicted prevalence across deciles of predicted probability; the mean absolute observed–predicted difference was 1.7 percentage points, and the largest absolute difference was 4.6 percentage points. Leverage and Cook’s distance checks did not identify observations that changed the interpretation of the key burden or limitation associations. An ordinal burden specification treating 2, 3, and ≥4 diagnosed conditions as an ordered three-level exposure was directionally consistent with the categorical model (aOR per higher burden category = 0.77, 95% CI 0.69–0.86), while any functional limitation remained strongly inversely associated with favorable mental-health self-appraisal (aOR = 0.32, 95% CI 0.26–0.39). The categorical burden specification was retained because it avoids imposing a linear trend across burden categories.

### 2.7. Missing Data and Sensitivity Analyses

Missingness in core covariates was low. Within the main analytic sample, missingness was 2.4% for usual source of care, 0.9% for education, 0.3% for limitation status, 0.05% for perceived physical health, and 0.02% for marital status. Because missingness was limited, variable-specific missingness was below 3%, and the fully adjusted complete-case model retained 5330 of 5523 respondents from the primary analytic sample, complete-case estimation was used for the primary multivariable analyses. This approach prioritized transparency and reproducibility from the public-use file and avoided introducing additional model-based assumptions through imputation when the expected gain in precision was limited. The two variables with the largest missingness, usual source of care and education, had missingness levels that were small relative to the analytic sample and were not the primary exposure or outcome variables. Therefore, complete-case exclusion was unlikely to materially change the main functional-limitation finding, although it could still affect the magnitude of socioeconomic or access-related gradients if item nonresponse was related to unmeasured vulnerability. Complete-case analysis assumes that exclusion due to missing covariate data did not materially alter the observed association structure after adjustment for measured characteristics. Potential selection related to item nonresponse therefore cannot be ruled out and is addressed explicitly in the Limitations Section.

Four sensitivity analyses were prespecified. First, the outcome was redefined more strictly as excellent/very good versus good/fair/poor. Second, perceived physical health was added to the model as an extension variable. Third, proxy-reporting status was added explicitly to the model. Fourth, the primary model was repeated after restricting the sample to respondents not reported by a proxy. Self-Administered Questionnaire mental-health scales such as K6 or PHQ-2 were not used in the primary model set because that would have required a separate adult-only SAQ-weighted design with a smaller effective sample and a different inferential target (Agency for Healthcare Research and Quality, 2025b).

### 2.8. Reporting Guideline and Reproducibility

This manuscript was prepared in accordance with the STROBE Statement for cross-sectional studies (von Elm et al., 2007). Variable coding decisions, sample-selection logic, weighting variables, model specifications, and sensitivity analyses are reported to facilitate reproduction from the public-use file. The reproducibility scripts were prepared and checked using R version 4.4 with the readr, dplyr, and survey packages, and Stata version 18.0 using base survey commands. Supplementary File S1 provides the source-data workbook underlying the manuscript tables and data-driven figures, Supplementary File S2 provides the high-resolution figure image files used in the manuscript, and Supplementary File S3 provides annotated R and Stata code (the reproducibility scripts were prepared and checked using R version 4.4 with the readr, dplyr, and survey packages, and Stata version 18.0 using base survey commands), software information, variable recoding rules, survey-design settings, model formulas, robustness specifications, and expected checkpoint outputs.

## 3. Results

### 3.1. Sample Selection and Analytic Population

**Figure 2:**
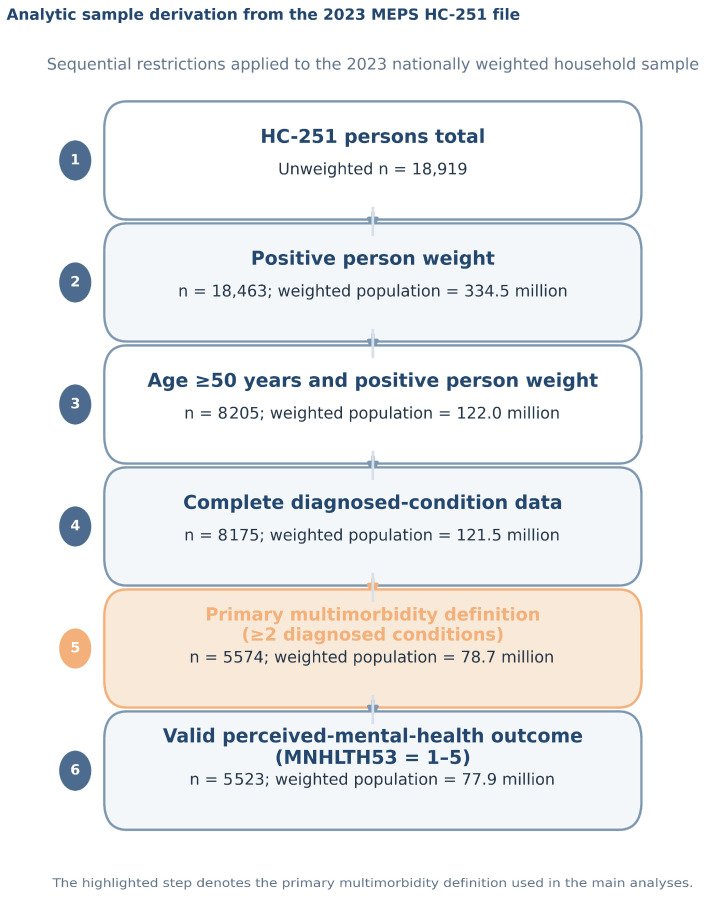
STROBE-style sample-selection flowchart for the 2023 HC-251 analytic sample.

Figure 2 presents the STROBE-style sample-selection process for the 2023 HC-251 analytic sample.

### 3.2. Weighted Sample Characteristics

The weighted analytic sample was 52.7% female and 47.3% male. By age, 40.6% were 50–64 years, 32.9% were 65–74 years, and 26.5% were aged ≥ 75 years. Most respondents were non-Hispanic White (71.1%), 55.8% were married, and 33.8% held a bachelor’s degree or higher. In socioeconomic terms, 46.0% were in the high-income category and 61.9% were not employed. With respect to health-system resources, 54.5% had any private insurance, 43.2% had public-only coverage, and 88.0% reported a usual source of care. In the need/functioning block, 39.2% had four or more diagnosed conditions and 39.9% reported at least one limitation. The full weighted descriptive profile is shown in Table 2.

**Figure 3:**
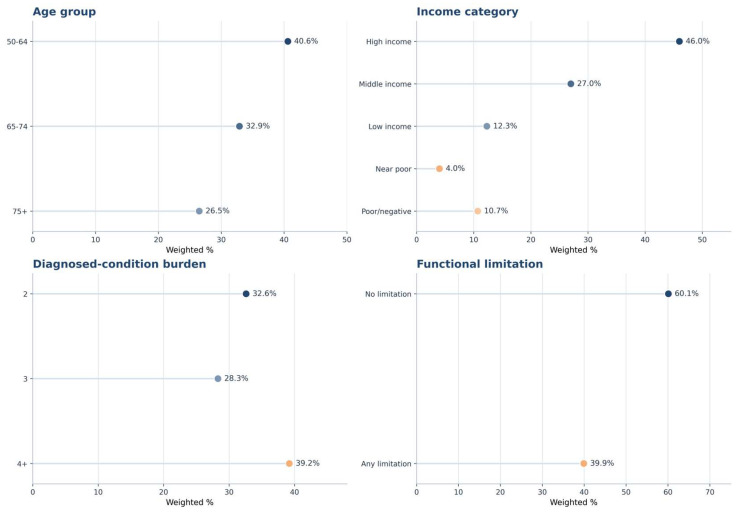
Weighted composition of the analytic sample across selected age, income, illness-burden, and limitation domains. Colors distinguish categories within each domain and are used to improve visual separation of the plotted weighted percentages.

Figure 3 complements Table 2 by highlighting the composition of the analytic population across four domains that are behaviorally informative: age, income, diagnosed-condition burden, and limitation status. The figure shows that the sample was not dominated by a single vulnerable subgroup. Instead, later-life multimorbidity coexisted with substantial heterogeneity in social position and functional burden, underscoring why enabling resources and need/functioning factors should be considered simultaneously.

### 3.3. Prevalence of Favorable Mental-Health Self-Appraisal

**Figure 4:**
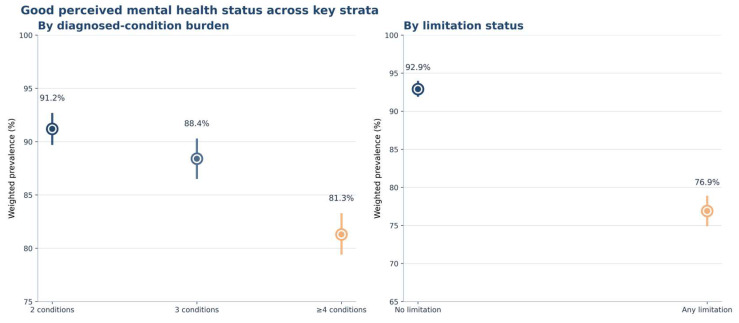
Weighted prevalence of favorable mental-health self-appraisal by diagnosed-condition burden and limitation status, with 95% confidence intervals. In the diagnosed-condition panel, colors distinguish the three burden categories (2, 3, and ≥4 diagnosed conditions). In the limitation-status panel, colors distinguish respondents without limitation from those with any limitation.

The weighted prevalence of favorable mental-health self-appraisal among U.S. adults aged ≥ 50 years with multimorbidity was 86.6% (95% CI 85.4–87.7). Figure 4 shows that prevalence declined monotonically as diagnosed-condition burden increased: 91.2% (95% CI 89.7–92.7) among those with two conditions, 88.4% (95% CI 86.5–90.3) among those with three conditions, and 81.3% (95% CI 79.4–83.3) among those with four or more diagnosed conditions.

Limitation status was associated with an even larger descriptive contrast. Favorable mental-health self-appraisal was reported by 92.9% (95% CI 91.9–94.0) of respondents without limitations, compared with 76.9% (95% CI 74.9–78.9) of those reporting any functional limitation. Taken together, the descriptive results suggested that both disease accumulation and constrained everyday functioning mattered, but that functioning had the steeper behavioral gradient.

### 3.4. Multivariable Associations

The complete-case fully adjusted core model included 5330 respondents, retaining 96.5% of the primary analytic sample. In the fully adjusted model, older age was associated with higher odds of favorable mental-health self-appraisal. Compared with adults aged 50–64 years, adults aged 65–74 years (aOR 1.52, 95% CI 1.17–1.98) and adults aged ≥ 75 years (aOR 1.79, 95% CI 1.36–2.36) had higher odds of a favorable outcome. Female sex was not independently associated with the outcome after full adjustment (aOR 0.89, 95% CI 0.74–1.08).

Several social and socioeconomic variables were independently associated with lower odds of favorable mental-health self-appraisal. Compared with non-Hispanic White respondents, Hispanic respondents (aOR 0.66, 95% CI 0.48–0.91) and non-Hispanic Asian respondents (aOR 0.54, 95% CI 0.35–0.84) had lower odds of a favorable outcome. Educational gradients were also evident: compared with respondents holding a bachelor’s degree or higher, those with less than a high school education had substantially lower odds (aOR 0.49, 95% CI 0.33–0.72), and those with some college education also had lower odds (aOR 0.72, 95% CI 0.53–0.98). Relative to the high-income group, low-income (aOR 0.67, 95% CI 0.48–0.93), near-poor (aOR 0.54, 95% CI 0.31–0.94), and poor/negative-income respondents (aOR 0.54, 95% CI 0.37–0.81) had lower odds. Non-employment was likewise associated with lower odds (aOR 0.60, 95% CI 0.45–0.79).

**Figure 5:**
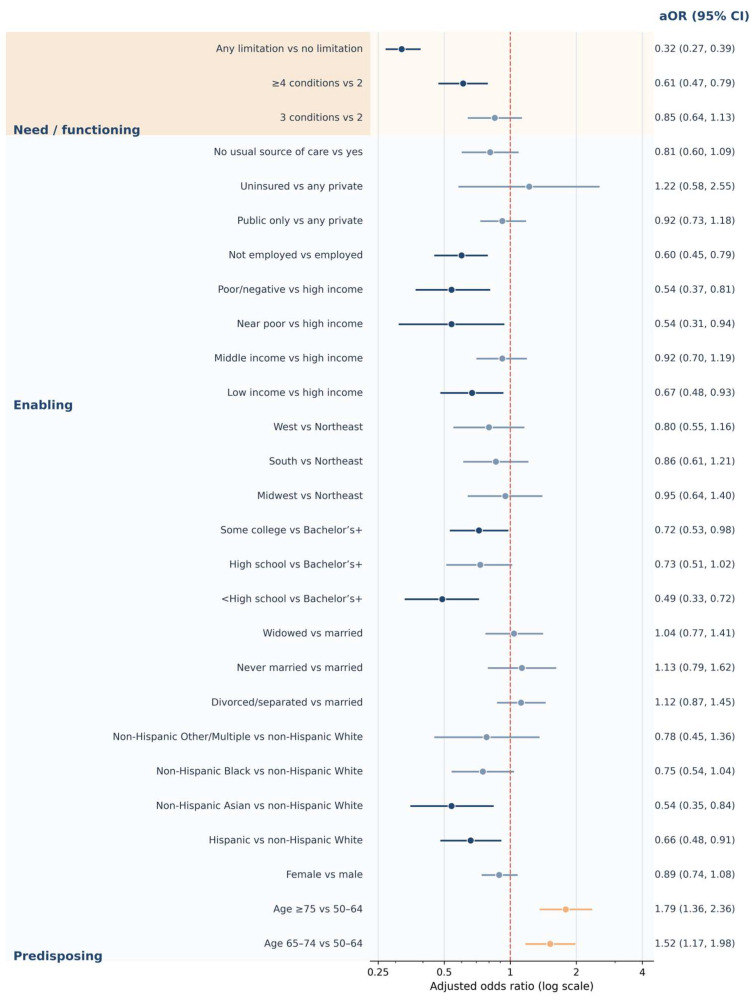
Fully adjusted correlates of favorable mental-health self-appraisal. Points show adjusted odds ratios (aORs), and horizontal lines show 95% confidence intervals. The red dashed vertical line indicates the null value (aOR = 1). Orange markers indicate statistically significant positive associations, dark-blue markers indicate statistically significant inverse associations, and light-blue markers indicate estimates whose 95% confidence intervals include the null value. Background shading distinguishes the predisposing, enabling, and need/functioning domains.

Condition burden and limitation status remained independently important after full adjustment. Compared with respondents who had two diagnosed conditions, those with three conditions did not differ significantly in the fully adjusted model (aOR 0.85, 95% CI 0.64–1.13), whereas respondents with four or more diagnosed conditions had significantly lower odds of favorable perceived mental-health self-appraisal (aOR 0.61, 95% CI 0.47–0.79). The strongest inverse association in the entire model was observed for any limitation (aOR 0.32, 95% CI 0.27–0.39). In contrast, marital status, region, insurance type, and usual source of care were not independently associated with the outcome after full adjustment. Table 3 and Figure 5 present the fully adjusted model estimates.

### 3.5. Marginal Interpretation of Burden and Limitation

**Figure 6:**
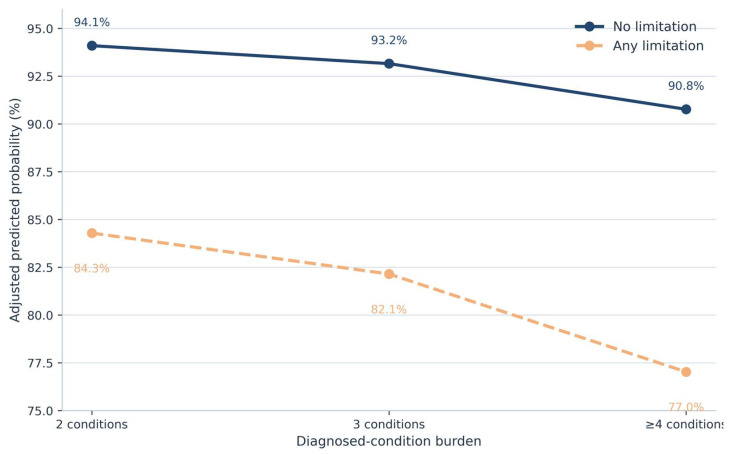
Adjusted predicted probabilities of favorable mental-health self-appraisal by diagnosed-condition burden and limitation status.

Figure 6 presents adjusted predicted probabilities derived from the fully adjusted core model. These estimates clarify the combined importance of burden and functioning. Among respondents without limitations, the adjusted predicted probability of favorable mental-health self-appraisal remained high even as disease burden increased: 94.1% for two conditions, 93.2% for three conditions, and 90.8% for four or more conditions. Among respondents with any limitation, the corresponding probabilities were consistently lower at every burden level (84.3%, 82.1%, and 77.0%, respectively).

Thus, the limitation gap was evident within each diagnosed-condition stratum and not only in the aggregate descriptive results. Model-based predicted probabilities help translate the odds-ratio findings into a more interpretable absolute-difference scale. They show that the behavioral burden associated with limitation status exceeded the incremental decline associated with moving from two to three diagnosed conditions and remained pronounced even among those with the heaviest disease burden. Because these predicted probabilities were derived from a cross-sectional regression model, they describe adjusted probability differences associated with diagnosed-condition burden and functional limitation status rather than causal effects of those characteristics.

### 3.6. Sensitivity Analyses

**Figure 7:**
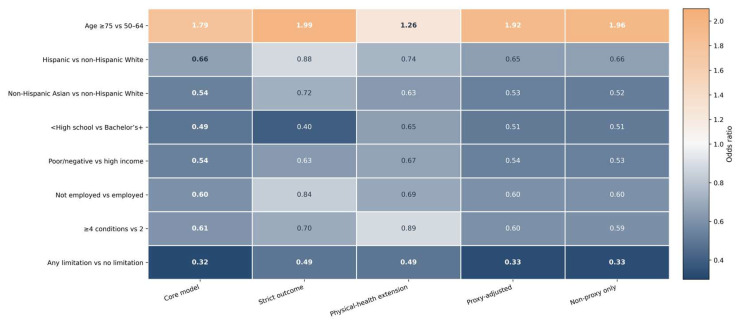
Robustness of selected associations across sensitivity analyses. Cell values are adjusted odds ratios. The color scale represents the magnitude of the odds ratio, with darker blue indicating lower odds ratios, lighter colors indicating values closer to 1, and orange indicating higher odds ratios.

Table 4 and Figure 7 summarize sensitivity analyses for selected adjusted associations with favorable mental-health self-appraisal. Across the stricter outcome definition, proxy-adjusted model, and non-proxy-restricted model, the direction of the key findings was preserved: older age remained positively associated with favorable self-appraisal, whereas lower education, lower income, non-employment, heavier disease burden, and any functional limitation remained inversely associated. The proxy-adjusted and non-proxy-restricted analyses were especially similar to the core model, suggesting that the main findings were not driven by household reporting context.

The physical-health extension model produced the largest attenuation. This attenuation was expected because perceived physical health and perceived mental health are both global evaluative ratings collected in the same household-interview context. In that model, several age- and burden-related estimates moved toward the null hypothesis, and the ≥4-condition coefficient was no longer statistically significant. However, attenuation should not be interpreted as substantive instability: the direction of the burden association remained inverse, and any functional limitation remained strongly associated with lower favorable mental-health self-appraisal (aOR 0.49, 95% CI 0.39–0.62). Overall, the sensitivity analyses indicate that the main functional-limitation pattern was robust to alternative outcome thresholds and reporting-context checks, while the physical-health extension model showed that part of the age and disease-burden association is shared with global physical-health appraisal.

## 4. Discussion

### 4.1. Principal Findings

This study estimated the weighted prevalence of favorable mental-health self-appraisal, identified associated behavioral, social, and functional characteristics, and compared the relative salience of diagnosed-condition burden and functional limitation in a nationally representative older-adult population living with multimorbidity. Three findings deserve emphasis. First, favorable mental-health self-appraisal remained common: 86.6% of U.S. adults aged ≥ 50 years with multimorbidity reported excellent, very good, or good perceived mental health. Second, favorable mental-health self-appraisal was not distributed evenly. Social disadvantage, heavier diagnosed-condition burden, and especially limitation status were associated with lower odds. Third, the burden gradient and the limitation gradient were not equivalent. In the fully adjusted model, the contrast between three versus two conditions was not statistically significant, whereas the contrast between four or more versus two conditions remained significant, and the limitation coefficient was by far the strongest inverse association.

The manuscript’s contribution is therefore not simply another description of multimorbidity prevalence. It reframes a national chronic-disease population through a behavioral-science question: under what functional and social conditions do older adults continue to appraise their mental health favorably? This shifts the emphasis from psychiatric deficit alone to adaptation, resources, and participation. Relative to prior MEPS-based behavioral sciences studies focused on pain, arthritis, pain interference, or opioid-treated pain (Axon & Chien, 2021; Awad & Axon, 2022; Axon & Kim, 2023; Axon & Agu, 2024), the present study extends the evidence to a broader multimorbidity population and directly compares diagnosis count with functional limitation as indicators of lived burden.

### 4.2. Functional Limitation as Lived Burden and Participation Restriction

The most behaviorally informative result is the prominence of limitation status. After full adjustment, respondents reporting any limitation had approximately one-third the odds of favorable mental-health self-appraisal compared with those without limitations. The adjusted predicted-probability analysis reinforced the same point: the limitation gap persisted within every disease-burden stratum. This pattern suggests that, in later life, the translation of illness into lived functional consequences may matter more for mental-health appraisal than diagnosis count alone. Two adults can each have multiple chronic conditions, yet the one who retains autonomy, mobility, and participation in valued roles may appraise overall mental health very differently from the one whose daily life is substantially constrained.

Several mechanisms may help explain this association. First, functional limitation can reduce autonomy in mobility, self-care, household tasks, and community participation, thereby narrowing opportunities for rewarding activity and social engagement. Second, limitations may undermine perceived control and self-efficacy, especially when everyday routines require assistance or become unpredictable. Third, functional impairment can increase treatment and care burden by making transportation, appointment attendance, medication management, and self-management behaviors more difficult. Fourth, limitations may intensify social isolation or role loss, particularly among older adults whose valued identities are tied to independence, work, caregiving, or community participation. These hypothesized pathways are consistent with the interpretation that functional status is not merely a clinical covariate but a lived behavioral burden through which multimorbidity becomes psychologically salient. Because the present analysis was cross-sectional and did not directly measure these mediating pathways, this interpretation should be treated as hypothesis-generating.

These proposed pathways are consistent with broader multimorbidity and quality-of-life research showing that functional impairment is not merely a downstream consequence of multimorbidity but also one of the major pathways through which chronic illness affects well-being and daily life (Ryan et al., 2015; Makovski et al., 2019; Calderón-Larrañaga et al., 2019). A recent meta-analysis on psychosocial determinants of functional independence among older adults further argues that social support, self-efficacy, and depressive symptoms are deeply intertwined with independence in activities of daily living (Goodarzi et al., 2024). In that sense, limitation status is not just a clinical control variable; it is a compact indicator of participation restriction, environmental barriers, and loss of behavioral repertoire.

A related implication is that favorable mental-health self-appraisal and functioning may interact bidirectionally. Positive affect can facilitate motivation, coping, adherence, and social engagement, while persistent functional loss can narrow opportunities for rewarding activity and threaten self-efficacy (Pressman & Cohen, 2005). The present cross-sectional design cannot disentangle those pathways, but the results underscore that functioning belongs at the center of older-adult multimorbidity research rather than at its analytic margins.

### 4.3. Age-Related Adaptation, Response Shift, and Socioemotional Prioritization

The age-group pattern observed in the fully adjusted core model should be interpreted cautiously. Adults aged 65–74 years and ≥75 years had higher adjusted odds of favorable mental-health self-appraisal than adults aged 50–64 years, but this cross-sectional association does not establish that aging itself causally improves mental health or produces more favorable self-appraisal. Rather, the pattern may reflect response shift, selective survival, cohort differences, altered expectations, accumulated coping strategies, socioemotional prioritization, reporting context, or other unmeasured factors rather than a direct age effect (Whitmore et al., 2022; van Loon et al., 2023; Sprangers & Schwartz, 1999). Because age group and mental-health self-appraisal were observed in the same survey period, the analysis cannot determine whether individuals became more favorable in their appraisal as they aged, whether adults with more favorable appraisal were more likely to remain in the civilian noninstitutionalized survey population, or whether unmeasured social, functional, or psychological factors contributed to the observed association (Jeste et al., 2025; Steptoe et al., 2015; Cosco et al., 2017). Future longitudinal work should directly test whether age-related differences in favorable mental-health self-appraisal are explained by response shift, social support, emotion regulation, selective survival, or cohort-specific experiences.

### 4.4. Socioeconomic Gradients and Structural Vulnerability

The socioeconomic findings point to a second major pathway: resources. Lower educational attainment, lower family income, and non-employment were each associated with lower odds of favorable mental-health self-appraisal. These contrasts should be interpreted cautiously. Global mental-health ratings may reflect cultural norms for self-appraisal, stigma, language, reference groups, and reporting context (Ahmad et al., 2014; McAlpine et al., 2018; Jylhä, 2009). Even so, the pattern cautions against viewing multimorbidity as socially neutral. The maintenance of favorable mental-health self-appraisal under chronic illness burden appears to be unequally distributed across social locations.

The present study also found lower odds of favorable mental-health self-appraisal among Hispanic and non-Hispanic Asian respondents relative to non-Hispanic White respondents. These contrasts should be interpreted cautiously. Global mental-health ratings may reflect cultural norms for self-appraisal, stigma, language, reference groups, and reporting context (Ahmad et al., 2014; McAlpine et al., 2018; Jylhä, 2009). Even so, the pattern cautions against viewing multimorbidity as socially neutral. The maintenance of favorable mental-health appraisal under chronic illness burden appears to be unequally distributed across social locations.

### 4.5. Interpreting a Single-Item Perceived Mental Health Outcome

Global perceived mental-health items are brief and policy-relevant, but they do not provide the same construct precision as multi-item scales of depression, psychological distress, or well-being (Ahmad et al., 2014; Fung et al., 2024; Hays et al., 2017; McAlpine et al., 2018; Lamers et al., 2011). The present analysis therefore interprets MNHLTH53 as a global appraisal measure rather than as a clinical diagnosis or multidimensional measure of positive mental health. Dichotomizing the five-level item improved interpretability and comparability with prior MEPS-based studies, but it inevitably reduced ordinal information and may have obscured differences between excellent, very good, and good appraisals (Ahmad et al., 2014; McAlpine et al., 2018).

Reporting heterogeneity is also possible because appraisal-based ratings may be shaped by cultural norms, reference groups, stigma, and proxy-reporting context (Sprangers & Schwartz, 1999; Jylhä, 2009). The sensitivity analyses partly addressed these concerns: the stricter outcome threshold, proxy adjustment, non-proxy restriction, and physical-health extension model all preserved the main functional-limitation pattern, although they cannot eliminate measurement limitations or reporting heterogeneity.

### 4.6. Implications for Health Psychology, Gerontology, and Service Design

The findings have practical implications for behavioral research and service design. First, screening and service-design efforts for older adults with multimorbidity should not focus exclusively on diagnosis counts. Functional limitation appears to be a more proximal correlate of favorable mental-health self-appraisal than diagnosis burden alone. These results identify mobility, role participation, self-management support, environmental adaptation, and access to rehabilitative or psychosocial resources as plausible priorities for future intervention research and service design, rather than proving that modifying these factors would causally change mental-health appraisal (Calderón-Larrañaga et al., 2019; Goodarzi et al., 2024).

Second, the study supports a strengths-based orientation in older-adult behavioral science. Research on multimorbidity often moves directly from chronic conditions to depression, distress, or decline. Those outcomes remain important, but they do not exhaust the mental-health landscape. A favorable-appraisal endpoint asks a different question: who is doing relatively well, and under what structural and functional conditions? That question is highly relevant to health psychology because it can inform interventions designed not only to reduce pathology but also to preserve autonomy, agency, coping, and well-being in people who are likely to remain medically complex (Jeste et al., 2025; Steptoe et al., 2015; Seligman & Csikszentmihalyi, 2000; Diener et al., 1999).

Third, the paper has implications for population monitoring and service access. Older adults frequently encounter barriers to formal mental-health care, including stigma, normalization of symptoms, practical access barriers, and uncertainty about where to seek help (Elshaikh et al., 2023). In that context, a brief global mental-health item may serve as an efficient signal that identifies who may merit more comprehensive assessment. The present study cannot determine whether such screening would improve care pathways, but it helps identify which older adults with multimorbidity appear least likely to report favorable mental-health self-appraisal and therefore may warrant closer attention.

### 4.7. Strengths, Limitations, and Future Directions

This study has several strengths. It used the newest publicly available full-year MEPS consolidated file (Agency for Healthcare Research and Quality, 2025b), adhered to HC-251 weighting and variance-estimation specifications (Agency for Healthcare Research and Quality, 2025a, 2025b), focused on an important older multimorbidity population, and applied multiple sensitivity analyses rather than relying on a single specification. It also used a conservative diagnosis-based multimorbidity definition intended to reduce construct ambiguity and foregrounded a favorable appraisal endpoint rather than restricting the analysis to poor mental health or distress. The manuscript was framed and reported in line with STROBE expectations for cross-sectional studies (von Elm et al., 2007).

Several limitations should also be acknowledged. First, the cross-sectional design precludes causal inference and prevents temporal ordering. Functional limitation may be associated with lower favorable mental-health self-appraisal, but lower appraisal, distress, or unmeasured psychological factors may also influence how respondents report limitation, perceived physical health, or perceived mental health. The associations should therefore be interpreted as descriptive and explanatory of population patterning rather than as evidence that functional limitation or age causally changes mental-health self-appraisal. Second, MEPS represents the U.S. civilian noninstitutionalized population, so the findings should not be generalized to older adults living in nursing homes, prisons, or other institutions (Agency for Healthcare Research and Quality, 2025b). This exclusion may systematically underrepresent older adults with the most severe functional, cognitive, or psychiatric impairment. Although the MEPS weight calibration addresses the civilian noninstitutionalized target population, the study’s estimates do not describe institutionalized older adults. Third, the study relied on household-interview reported measures. MNHLTH53 is a single global appraisal item rather than a diagnostic interview, symptom-severity scale, psychological-distress scale, or multidimensional well-being measure. This makes the item brief and policy-relevant, but it limits construct precision. In addition, chronic-condition and limitation indicators may be affected by recall, diagnosis awareness, access to care, and proxy-reporting context. Possible misclassification of chronic conditions could underestimate or distort diagnosed-condition burden, especially among respondents with limited care access or lower diagnosis awareness. Although proxy adjustment and non-proxy restriction were reassuring, reporting heterogeneity cannot be eliminated.

Fourth, selection bias related to missing covariate data remains possible because the fully adjusted model used complete-case estimation. The extent of missingness was small, with variable-specific missingness below 3% and 96.5% of the primary analytic sample retained in the complete-case model, so substantial loss of information is unlikely. Nevertheless, if missingness in usual source of care or education was related to unmeasured socioeconomic vulnerability, care disconnection, or mental-health self-appraisal after adjustment for observed covariates, the adjusted socioeconomic or access-related associations could still be biased. The main functional-limitation association is less likely to be driven by item missingness because limitation missingness was very low, but selection related to complete-case estimation cannot be ruled out.

Fifth, the primary multimorbidity definition was intentionally conservative and diagnosis-based. Although this improves construct clarity, it may underestimate disease burden relative to broader symptom-inclusive, respiratory-symptom, post-viral, or conditions-file-based definitions (Huntley et al., 2012; Stirland et al., 2020). The exclusion of joint pain, chronic bronchitis, COVID-19, and long-COVID indicators improves stability of the primary diagnosis-count construct but reduces comparability with broader multimorbidity definitions. Finally, Self-Administered Questionnaire mental-health scales such as K6 or PHQ-2 were not used in the main analyses because that would have required a separate adult-only SAQ-weighted design with a smaller effective sample and a different inferential target (Agency for Healthcare Research and Quality, 2025b). Future research should test whether the same functional gradient appears when multimorbidity is defined using linked conditions files, severity-weighted burden indices, ordinal appraisal models, or adult-only distress/well-being scales with their own design weights.

## 5. Conclusions

In this nationally representative sample of U.S. civilian noninstitutionalized adults aged ≥ 50 years with multimorbidity, favorable mental-health self-appraisal remained common, but it was socially and functionally patterned. Older age was associated with higher odds of favorable self-appraisal, whereas social disadvantage, heavier diagnosed-condition burden, and especially any functional limitation were associated with lower odds. The magnitude and consistency of the limitation association suggest that lived functional burden may be a more behaviorally salient correlate of mental-health self-appraisal than diagnosis count alone.

These findings support a strengths-based health-psychology perspective on older-adult multimorbidity and highlight the value of examining who maintains favorable mental-health self-appraisal under chronic-disease burden. For behavioral science, the practical implication is that participation, autonomy, and functional independence should be considered alongside diagnosis counts when identifying older adults with multimorbidity who may be less likely to report favorable self-appraisal. Because the study was cross-sectional and relied on household-interview reported measures, including a single global appraisal item and respondent-reported chronic-condition and limitation indicators, the findings should be interpreted as associative rather than causal.

## Figures and Tables

**Table 1:** Analysis-variable map and operational definitions.

Outcome	Favorable Mental-Health Self-Appraisal	MNHLTH53 (PE20)	Excellent/Very Good/Good vs. Fair/Poor	Primary Behavioral Self-Appraisal Endpoint
Main exposure/sample restriction	Primary multimorbidity definition	HIBPDX, CHDDX/ANGIDX/MIDX/OHRTDX, STRKDX, EMPHDX, CHOLDX, CANCERDX, ARTHDX, DIABDX_M18, ASTHDX	≥2 diagnosed conditions; heart disease counted as one composite domain	Conservative diagnosis-based multimorbidity construct
Predisposing	Age group	AGE23X	50–64/65–74/≥75 years	Life-stage differences in appraisal/adaptation
Predisposing	Sex	SEX	Male/female	Standard demographic covariate
Predisposing	Race/ethnicity	RACETHX/HISPANX-derived edited categories	Hispanic; non-Hispanic White; non-Hispanic Black; non-Hispanic Asian; non-Hispanic other/multiple	Sociodemographic patterning and reporting context
Predisposing	Marital status	MARRY23X	Married; divorced/separated; never married; widowed	Social role and support context
Predisposing	Education	HIDEG/highest degree variable	<High school; high school; some college; bachelor’s+	Human capital/socioeconomic position
Predisposing	Region	REGION23	Northeast; Midwest; South; West	Geographic context
Enabling	Family poverty category	POVCAT23	High; middle; low; near poor; poor/negative	Economic resources
Enabling	Employment status	EMPST53H	Employed vs. not employed	Role participation and financial security
Enabling	Insurance coverage	Any private/public only/uninsured	Collapsed from annual insurance indicators	Health-care financing
Enabling	Usual source of care	HAVEUS42	Yes vs. no	Care continuity/access
Need/functioning	Condition burden	Primary diagnosed-condition count	2/3/≥4 conditions	Burden gradient within multimorbidity
Need/functioning	Any limitation	ANYLMI23	Any limitation vs. none	Burden gradient within multimorbidity
Extension only	Perceived physical health status	RTHLTH53	Excellent/very good/good vs. fair/poor	Functional status/lived behavioral burden
Sensitivity only	Proxy reporting status	PROXY53	Included as adjustment and non-proxy restriction	Assesses reporting heterogeneity

**Table 2:** Weighted characteristics of U.S. adults aged ≥ 50 years with multimorbidity in the 2023 MEPS analytic sample (*N* = 5523).

Section	Characteristic	Unweighted n	Weighted %
Age group, years	50–64	1965	40.6
	65–74	1923	32.9
	75+	1635	26.5
Sex	Female	3036	52.7
	Male	2487	47.3
Race/ethnicity	Hispanic	666	10.6
	Non-Hispanic Asian	202	4.3
	Non-Hispanic Black	744	11.6
	Non-Hispanic Other/Multiple	135	2.5
	Non-Hispanic White	3776	71.1
Marital status	Divorced/Separated	1181	19.8
	Married	2833	55.8
	Never married	538	8.8
	Widowed	970	15.5
Education	<High school	765	10.6
	Bachelor’s+	1792	33.8
	High school	1705	31.9
	Some college	1214	23.7
Region	Midwest	1175	21.2
	Northeast	936	17.7
	South	2143	40.1
	West	1269	21.0
Family income as % of federal poverty line	High income	2205	46.0
	Low income	721	12.3
	Middle income	1467	27.0
	Near poor	288	4.0
	Poor/negative	842	10.7
Employment status	Employed	1805	38.1
	Not employed	3718	61.9
Insurance	Any private	2735	54.5
	Public only	2680	43.2
	Uninsured	108	2.3
Usual source of care	No	598	12.0
	Yes	4790	88.0
Diagnosed-condition burden	2	1690	32.6
	3	1536	28.3
	4+	2297	39.2
Any limitation	Any limitation	2404	39.9
	No limitation	3103	60.1

**Table 3:** Fully adjusted survey-weighted logistic regression model for favorable mental-health self-appraisal (complete-case *n* = 5330).

Characteristic	aOR (95% CI)	*p*
Age 65–74 vs. 50–64	1.52 (1.17, 1.98)	0.002
Age ≥ 75 vs. 50–64	1.79 (1.36, 2.36)	<0.001
Female vs. male	0.89 (0.74, 1.08)	0.237
Hispanic vs. non-Hispanic White	0.66 (0.48, 0.91)	0.012
Non-Hispanic Asian vs. non-Hispanic White	0.54 (0.35, 0.84)	0.006
Non-Hispanic Black vs. non-Hispanic White	0.75 (0.54, 1.04)	0.081
Non-Hispanic Other/Multiple vs. non-Hispanic White	0.78 (0.45, 1.36)	0.374
Divorced/separated vs. married	1.12 (0.87, 1.45)	0.374
Never married vs. married	1.13 (0.79, 1.62)	0.490
Widowed vs. married	1.04 (0.77, 1.41)	0.778
<High school vs. bachelor’s+	0.49 (0.33, 0.72)	<0.001
High school vs. bachelor’s+	0.73 (0.51, 1.02)	0.068
Some college vs. bachelor’s+	0.72 (0.53, 0.98)	0.036
Midwest vs. Northeast	0.95 (0.64, 1.40)	0.778
South vs. Northeast	0.86 (0.61, 1.21)	0.376
West vs. Northeast	0.80 (0.55, 1.16)	0.238
Low income vs. high income	0.67 (0.48, 0.93)	0.018
Middle income vs. high income	0.92 (0.70, 1.19)	0.511
Near poor vs. high income	0.54 (0.31, 0.94)	0.028
Poor/negative vs. high income	0.54 (0.37, 0.81)	0.003
Not employed vs. employed	0.60 (0.45, 0.79)	<0.001
Public only vs. any private	0.92 (0.73, 1.18)	0.520
Uninsured vs. any private	1.22 (0.58, 2.55)	0.603
No usual source of care vs. yes	0.81 (0.60, 1.09)	0.167
3 conditions vs. 2	0.85 (0.64, 1.13)	0.268
≥4 conditions vs. 2	0.61 (0.47, 0.79)	<0.001
Any limitation vs. no limitation	0.32 (0.27, 0.39)	<0.001

**Table 4:** Summary of key sensitivity analyses for selected characteristics.

Characteristic	Core Model	Strict Outcome	Physical-Health Extension	Proxy-Adjusted	Non-Proxy Only
Age ≥ 75 vs. 50–64	1.79 (1.36, 2.36)	1.99 (1.60, 2.47)	1.26 (0.93, 1.71)	1.92 (1.46, 2.53)	1.96 (1.48, 2.58)
Hispanic vs. non-Hispanic White	0.66 (0.48, 0.91)	0.88 (0.67, 1.16)	0.74 (0.52, 1.04)	0.65 (0.47, 0.90)	0.66 (0.48, 0.91)
Non-Hispanic Asian vs. non-Hispanic White	0.54 (0.35, 0.84)	0.72 (0.46, 1.13)	0.63 (0.40, 0.98)	0.53 (0.34, 0.83)	0.52 (0.34, 0.81)
<High school vs. bachelor’s+	0.49 (0.33, 0.72)	0.40 (0.31, 0.52)	0.65 (0.44, 0.97)	0.51 (0.35, 0.75)	0.51 (0.35, 0.74)
Poor/negative vs. high income	0.54 (0.37, 0.81)	0.63 (0.49, 0.82)	0.67 (0.44, 1.04)	0.54 (0.36, 0.81)	0.53 (0.36, 0.80)
Not employed vs. employed	0.60 (0.45, 0.79)	0.84 (0.70, 1.00)	0.69 (0.52, 0.91)	0.60 (0.46, 0.80)	0.60 (0.46, 0.80)
≥4 conditions vs. 2	0.61 (0.47, 0.79)	0.70 (0.59, 0.83)	0.89 (0.66, 1.19)	0.60 (0.46, 0.78)	0.59 (0.46, 0.78)
Any limitation vs. no limitation	0.32 (0.27, 0.39)	0.49 (0.43, 0.56)	0.49 (0.39, 0.62)	0.33 (0.27, 0.40)	0.33 (0.27, 0.41)

## Data Availability

The data used in this study are publicly available from the Agency for Healthcare Research and Quality through the 2023 Medical Expenditure Panel Survey (MEPS) Full Year Consolidated Data File (HC-251) and its accompanying documentation. Supplementary File S1 provides the source data underlying the manuscript tables and data-driven figures. Supplementary File S2 provides the figure image files. Supplementary File S3 provides the annotated analytic code and reproducibility documentation used to recreate the study sample, variable recoding, survey design settings, model specifications, and robustness analyses.

## Supplementary Materials

The following supporting information can be downloaded at: https://www.mdpi.com/article/10.3390/bs16060841/s1, Supplementary File S1: source-data workbook underlying the manuscript tables and data-driven figures; Supplementary File S2: high-resolution figure image files used in the manuscript; Supplementary File S3: reproducibility materials, including annotated R and Stata code, software information, variable recoding rules, survey-design settings, model formulas, robustness specifications, and expected checkpoint outputs.
