# Supplementary material for: Functional Limitation and Favorable Mental-Health Self-Appraisal Among U.S. Adults Aged 50 Years or Older with Multimorbidity: A Behavioral-Science Analysis of the 2023 Medical Expenditure Panel Survey
Source: Behav Sci (Basel). 2026 May 22;16(6):841. doi: 10.3390/bs16060841 (PMC13295625; doi:10.3390/bs16060841)
Supplement: Supplementary file 1 [file behavsci-16-00841-s001.zip › STROBE_Checklist_Behavioral_Sciences_MEPS2023_20260420.pdf]

STROBE Statement Checklist for Cross-Sectional Studies

Manuscript: Functional Limitation and Favorable Mental-Health Appraisal among U.S. Adults Aged 50 Years or Older with Multimorbidity: A Behavioral-Science Analysis of the 2023 Medical Expenditure Panel Survey

Completed for submission to Behavioral Sciences. Section references correspond to the clean submission manuscript dated 20 April 2026.

Section	Item	STROBE Recommendation	Location in Manuscript
Title and abstract	1a	Indicate the study design with a commonly used term in the title or abstract.	Title; Abstract
Title and abstract	1b	Provide an informative and balanced summary of what was done and what was found.	Abstract
Introduction: Background/rationale	2	Explain the scientific background and rationale for the investigation.	Introduction
Introduction: Objectives	3	State specific objectives, including prespecified questions or hypotheses.	End of Introduction
Methods: Study design	4	Present key elements of study design early in the paper.	2.1 Study Design and Data Source
Methods: Setting	5	Describe setting, locations, and relevant dates, including data collection periods.	2.1 Study Design and Data Source
Methods: Participants	6	Give eligibility criteria, sources, and methods of selection of participants.	2.2 Study Population and Sample Selection; Figure 2
Methods: Variables	7	Clearly define outcomes, exposures, predictors, potential confounders, and effect modifiers.	2.3–2.5; Table 1
Methods: Data sources/measurement	8	For each variable of interest, give sources of data and details of assessment methods.	2.3–2.5; Table 1; Supplementary File S3
Methods: Bias	9	Describe efforts to address potential sources of bias.	2.6–2.7; 4.5 Interpreting a Single-Item Outcome; 4.7 Limitations
Methods: Study size	10	Explain how the study size was arrived at.	2.2; Figure 2
Methods: Quantitative variables	11	Explain how quantitative variables were handled in the analyses.	2.3–2.6; Table 1; Supplementary File S3
Methods: Statistical methods	12a	Describe all statistical methods, including those used to control for confounding.	2.6 Statistical Analysis
Methods: Statistical methods	12b	Describe methods used to examine subgroups and interactions, if applicable.	2.6; 2.7; no interaction model was prespecified
Methods: Statistical methods	12c	Explain how missing data were addressed.	2.7 Missing Data and Sensitivity Analyses
Methods: Statistical methods	12d	If applicable, describe analytical methods taking account of sampling strategy.	2.6 Statistical Analysis
Methods: Statistical methods	12e	Describe sensitivity analyses.	2.7; 3.6 Sensitivity Analyses; Table 4; Figure 7
Results: Participants	13a	Report numbers at each stage of study selection.	3.1; Figure 2
Results: Participants	13b	Give reasons for non-participation at each stage, when applicable.	3.1; Figure 2; secondary public-use dataset
Results: Participants	13c	Consider use of a flow diagram.	Figure 2
Results: Descriptive data	14a	Give characteristics of study participants and information on exposures and potential confounders.	3.2; Table 2; Figure 3
Results: Descriptive data	14b	Indicate number of participants with missing data for each variable of interest.	2.7; Supplementary File S3
Results: Descriptive data	14c	Summarize follow-up time, if applicable.	Not applicable: cross-sectional analysis
Results: Outcome data	15	Report numbers of outcome events or summary measures.	3.3; Figure 4
Results: Main results	16a	Give unadjusted and adjusted estimates and their precision.	3.3–3.5; Tables 3–4; Figures 4–6
Results: Main results	16b	Report category boundaries when continuous variables were categorized.	2.3–2.5; Table 1
Results: Main results	16c	Translate relative risk into absolute risk for a meaningful time period when relevant.	3.5; Figure 6 reports adjusted predicted probabilities
Results: Other analyses	17	Report other analyses done, including sensitivity analyses.	3.6; Table 4; Figure 7
Discussion: Key results	18	Summarize key results with reference to study objectives.	4.1 Principal Findings
Discussion: Limitations	19	Discuss limitations, sources of potential bias or imprecision, and direction/magnitude where relevant.	4.7 Strengths, Limitations, and Future Directions
Discussion: Interpretation	20	Give a cautious overall interpretation considering objectives, limitations, multiplicity, and evidence from similar	4.2–4.7; 5 Conclusions

		studies.	
Discussion: Generalizability	21	Discuss the generalizability of the results.	4.7 Strengths, Limitations, and Future Directions
Other information: Funding	22	Give the source of funding and role of funders.	Funding; Conflicts of Interest
