# Supplementary material for: Functional Limitation and Favorable Mental-Health Self-Appraisal Among U.S. Adults Aged 50 Years or Older with Multimorbidity: A Behavioral-Science Analysis of the 2023 Medical Expenditure Panel Survey
Source: Behav Sci (Basel). 2026 May 22;16(6):841. doi: 10.3390/bs16060841 (PMC13295625; doi:10.3390/bs16060841)
Supplement: Supplementary file 1 [file behavsci-16-00841-s001.zip › Supplementary_File_S3_Reproducibility_Materials/Supplementary_File_S3_Reproducibility_Materials.pdf]

Supplementary File S3. Reproducibility Materials

Manuscript title

Functional Limitation and Favorable Mental-Health Appraisal among U.S. Adults Aged 50 Years or Older with Multimorbidity: A Behavioral-Science Analysis of the 2023 Medical Expenditure Panel Survey

Purpose

This file documents the public-use analytic workflow used to reproduce the study sample, variable recoding, survey-design settings, model specifications, and robustness checks from the 2023 MEPS Full Year Consolidated Data File (HC-251). The file accompanies two annotated code scripts:

1. behavioral_sciences_meps2023_reproducibility_r.R
2. behavioral_sciences_meps2023_reproducibility_stata.do

Data source

The analyses use the 2023 Medical Expenditure Panel Survey (MEPS) Full Year Consolidated Data File, HC-251, distributed by the Agency for Healthcare Research and Quality (AHRQ). The public-use ASCII file is h251.dat. Official variable positions and import statements are available from AHRQ for R, Stata, and SAS.

Required input files

1. h251.dat
2. The code files supplied in this Supplementary File S3

Software information for the supplied scripts

1. R implementation: written for R 4.4 or later using readr, dplyr, and survey
2. Stata implementation: written for Stata 18 or later using base survey commands

Analytic population

The study population is restricted to adults aged 50 years or older with a positive full-year person weight (PERWT23F > 0). The primary diagnosed-condition multimorbidity count is then constructed from nine diagnosis domains:

1. High blood pressure (HIBPDX)
2. Composite heart disease, defined as any of CHDDX, ANGIDX, MIDX, or OHRTDX = yes
3. Stroke (STRKDX)
4. Emphysema (EMPHDX)
5. High cholesterol (CHOLDX)
6. Cancer (CANCERDX)
7. Arthritis (ARTHDX)
8. Diabetes (DIABDX_M18)
9. Asthma (ASTHDX)

Heart-disease composite rule

Heart disease is coded present when any of CHDDX, ANGIDX, MIDX, or OHRTDX equals yes. A respondent is treated as having complete information for the heart-disease composite if:

1. At least one of the four component variables equals yes, or
2. All four component variables equal no

This rule avoids discarding respondents who clearly have heart disease even if one of the component items is missing.

Condition-data completeness rule

Respondents are retained for multimorbidity counting when:

1. The heart-disease composite is complete under the rule above, and
2. HIBPDX, STRKDX, EMPHDX, CHOLDX, CANCERDX, ARTHDX, DIABDX_M18, and ASTHDX each take valid yes/no values

Primary multimorbidity definition

Primary diagnosed-condition multimorbidity is defined as a diagnosed-condition count >= 2 across the nine domains listed above.

Primary outcome

Favorable perceived mental-health appraisal is derived from MNHLTH53:

1. Favorable = excellent / very good / good
2. Reference category = fair / poor

Sensitivity outcome

The stricter sensitivity outcome defines favorable appraisal as:

1. Favorable = excellent / very good
2. Reference category = good / fair / poor

Operational recoding used in the core model

1. Age group from AGE23X:

   50-64, 65-74, >=75 years

2. Sex from SEX:

male, female

3. Race/ethnicity from RACETHX:

Hispanic

Non-Hispanic White

Non-Hispanic Black

Non-Hispanic Asian

Non-Hispanic Other/Multiple

4. Marital status from MARRY23X:

Married

Divorced/Separated (codes divorced and separated combined)

Never married

Widowed

5. Education from EDUCYR:

<High school = 0-11 years

High school = 12 years

Some college = 13-15 years

Bachelor's+ = 16-17 years

6. Census region from REGION23:

Northeast, Midwest, South, West

7. Family income as percent of federal poverty line from POVCAT23:

High income

Middle income

Low income

Near poor

Poor/negative

8. Employment status from EMPST53H:

Employed = employed at interview date or job to return to

Not employed = not employed at interview date / not employed during round

9. Insurance from INSCOV23:

Any private

Public only

Uninsured

10. Usual source of care from HAVEUS42:

yes, no

11. Diagnosed-condition burden:

2 conditions

3 conditions

>=4 conditions

12. Any limitation from ANYLMI23:

any limitation, no limitation

Extension and robustness variables

1. Perceived physical health from RTHLTH53:
   good = excellent / very good / good
   fair/poor = fair / poor

2. Proxy-reporting status from PROXY53:
   RU member respondent
   proxy respondent

Survey-design settings

All person-level analyses use:

1. Weight: PERWT23F
2. Strata: VARSTR
3. Primary sampling unit: VARPSU
4. Variance estimation: Taylor-series linearization

Because the primary analysis does not use Self-Administered Questionnaire variables, SAQ weights are not used.

Missing-data handling

Primary multivariable models use complete-case estimation across the modeled covariates. In the manuscript-aligned implementation:

1. Main analytic sample with valid MNHLTH53: n = 5523
2. Core-model complete-case sample: n = 5330

3. Physical-health extension sample: n = 5327

4. Proxy-adjusted sample: n = 5330

5. Non-proxy complete-case sample: n = 5287

Model specifications

Model 1: predisposing block only

logit(Pr[Y = 1]) = beta0 + age group + sex + race/ethnicity + marital status + education + region

Model 2: predisposing + enabling

logit(Pr[Y = 1]) = Model 1 + poverty category + employment status + insurance + usual source of care

Model 3: core model

logit(Pr[Y = 1]) = Model 2 + diagnosed-condition burden + any limitation

Sensitivity model A: stricter outcome

Same covariates as Model 3, but Y = 1 only for excellent/very good mental-health ratings

Sensitivity model B: physical-health extension

Model 3 + perceived physical health

Sensitivity model C: proxy-adjusted

Model 3 + proxy-reporting status

Sensitivity model D: non-proxy restriction

Model 3 estimated after restricting to PROXY53 = RU member respondent

Expected reproducibility checkpoints

The supplied scripts should reproduce the following manuscript checkpoints from the public-use file:

1. HC-251 persons total = 18,919
2. Positive person weight = 18,463
3. Age >=50 and positive weight = 8,205
4. Complete diagnosed condition data = 8,175
5. Multimorbidity (>=2 diagnosed conditions) = 5,574
6. Valid MNHLTH53 (1-5) = 5,523
7. Weighted analytic population = 77,867,400.97
8. Weighted prevalence of favorable perceived mental-health appraisal = 86.6%
9. Weighted prevalence by burden:
   - 2 conditions = 91.2%
   - 3 conditions = 88.4%
   - >=4 conditions = 81.3%
10. Weighted prevalence by limitation:
    - no limitation = 92.9%
    - any limitation = 76.9%
11. Core-model complete-case n = 5,330

Key manuscript coefficients for comparison after model fitting are listed in reproducibility_checkpoints.txt.

Files included in this Supplementary File S3 folder

1. Supplementary_File_S3_Reproducibility_Materials.txt
2. behavioral_sciences_meps2023_reproducibility_r.R
3. behavioral_sciences_meps2023_reproducibility_stata.do
4. reproducibility_checkpoints.txt

Suggested manuscript-facing wording

Supplementary Materials:

Supplementary File S3: Reproducibility materials, including annotated R and Stata code, software information, variable recoding rules, survey-design settings, model formulas, and expected checkpoint outputs.

Data Availability Statement:

The data used in this study are publicly available from the Agency for Healthcare Research and Quality through the 2023 Medical Expenditure Panel Survey (MEPS) Full Year Consolidated Data File (HC-251) and its accompanying documentation. Supplementary File S1 provides the source data underlying the manuscript tables and data-driven figures. Supplementary File S2 provides the figure image files. Supplementary File S3 provides the annotated analytic code and reproducibility documentation used to recreate the study sample, variable recoding, survey design settings, model specifications, and robustness analyses.
