# Supplementary figures and images for: Functional Limitation and Favorable Mental-Health Self-Appraisal Among U.S. Adults Aged 50 Years or Older with Multimorbidity: A Behavioral-Science Analysis of the 2023 Medical Expenditure Panel Survey
Source: Behav Sci (Basel). 2026 May 22;16(6):841. doi: 10.3390/bs16060841 (PMC13295625; doi:10.3390/bs16060841)

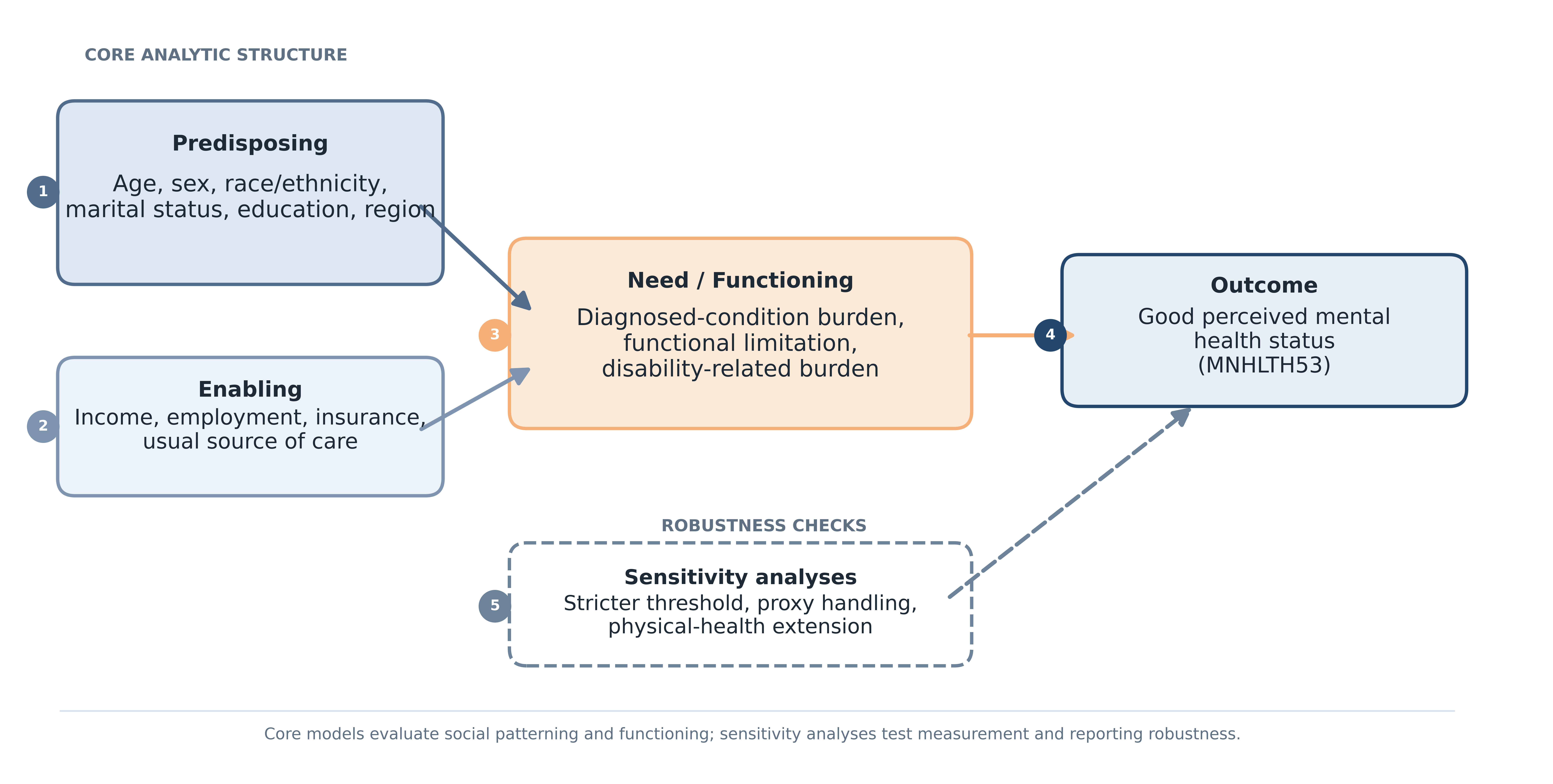

Supplement: Supplementary file 1 [file behavsci-16-00841-s001.zip › Supplementary_File_S2_Figures/Figure_1.png]

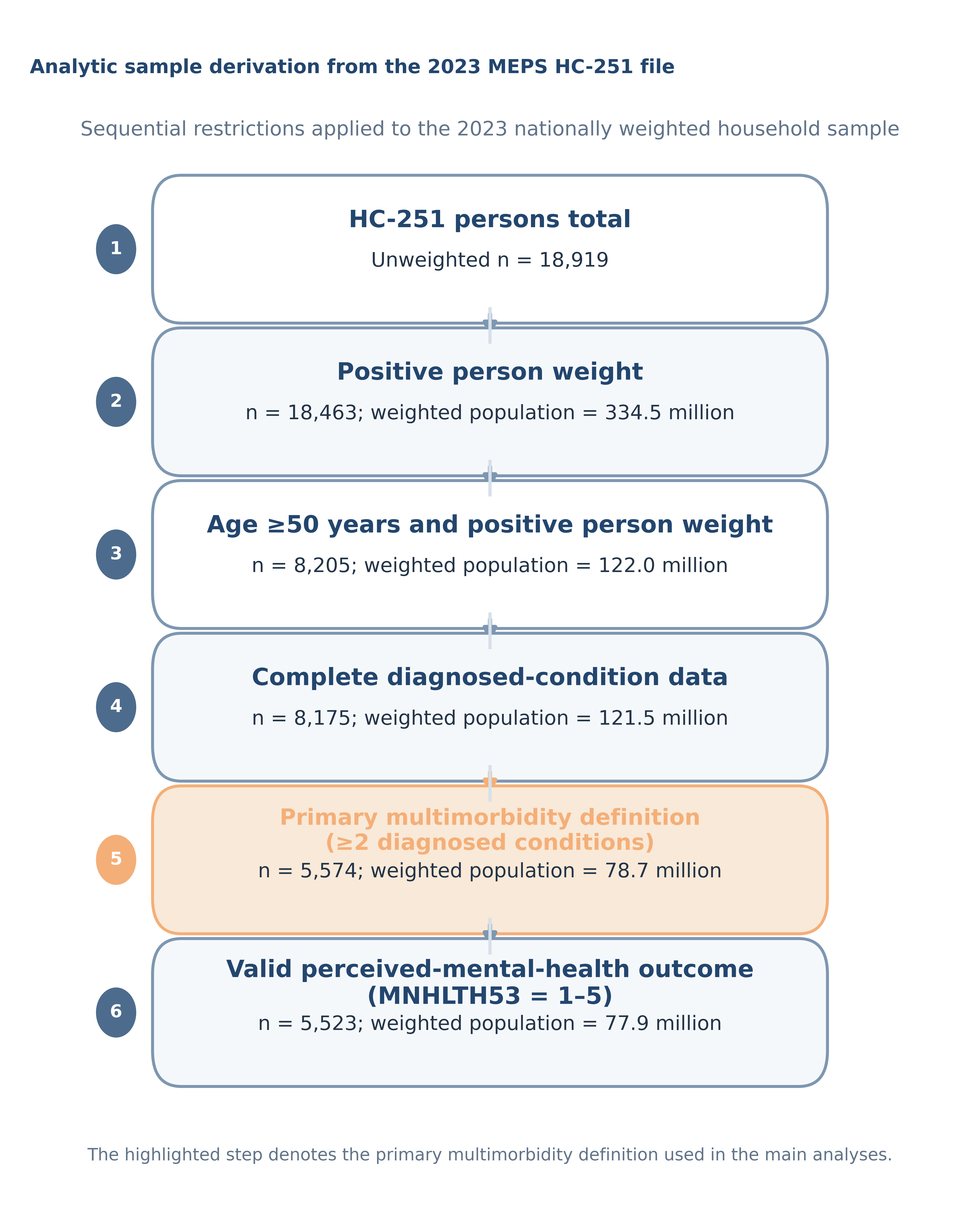

Supplement: Supplementary file 1 [file behavsci-16-00841-s001.zip › Supplementary_File_S2_Figures/Figure_2.png]

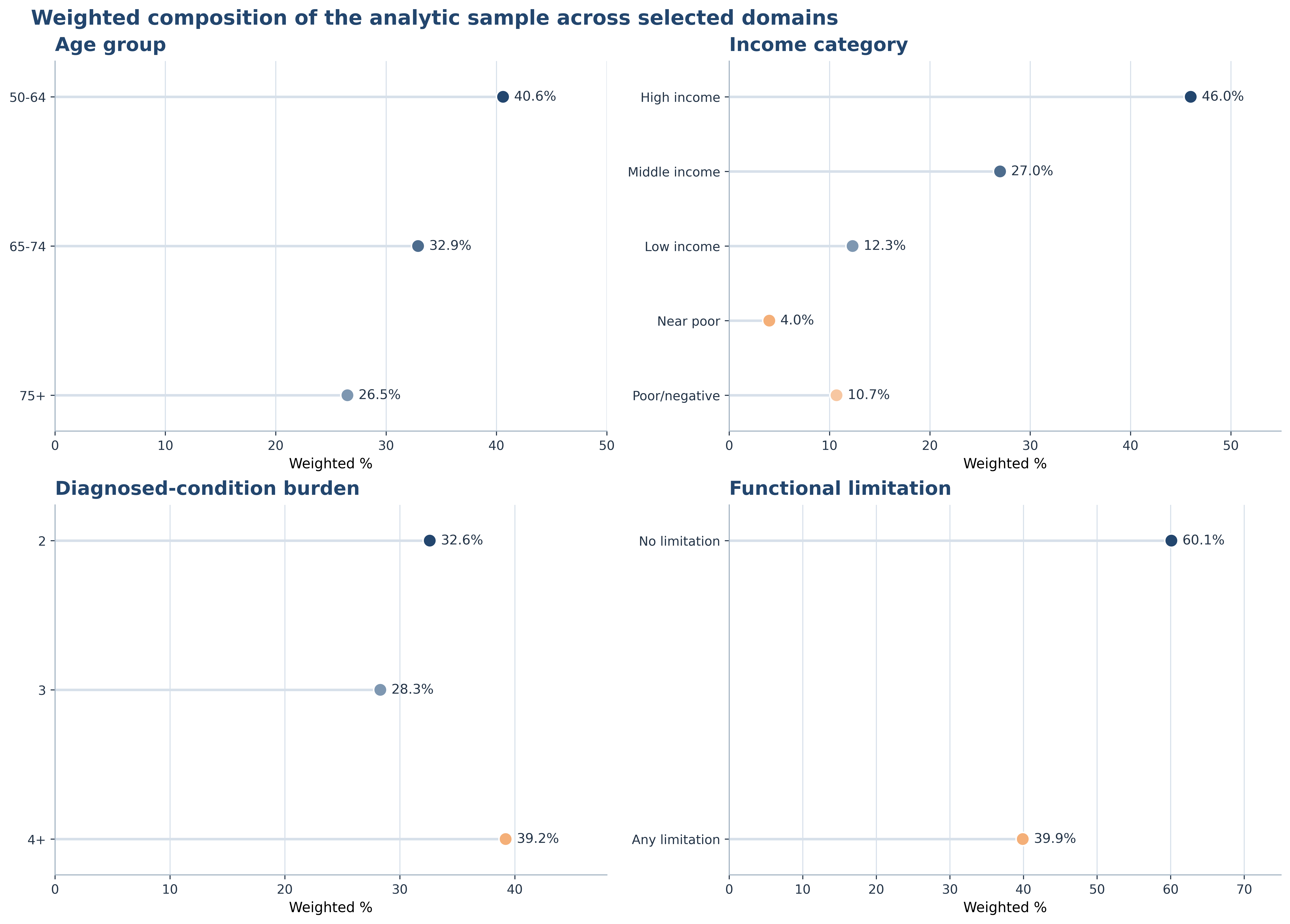

Supplement: Supplementary file 1 [file behavsci-16-00841-s001.zip › Supplementary_File_S2_Figures/Figure_3.png]

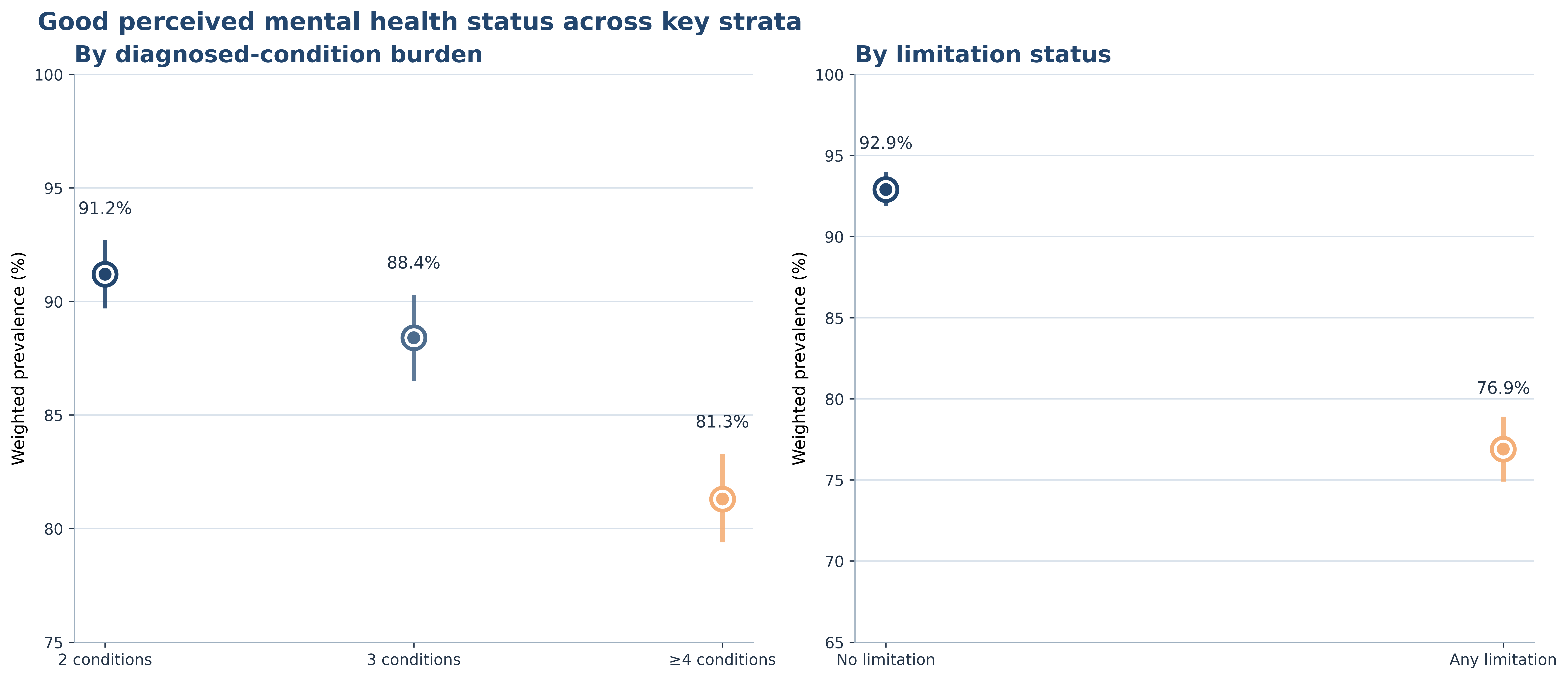

Supplement: Supplementary file 1 [file behavsci-16-00841-s001.zip › Supplementary_File_S2_Figures/Figure_4.png]

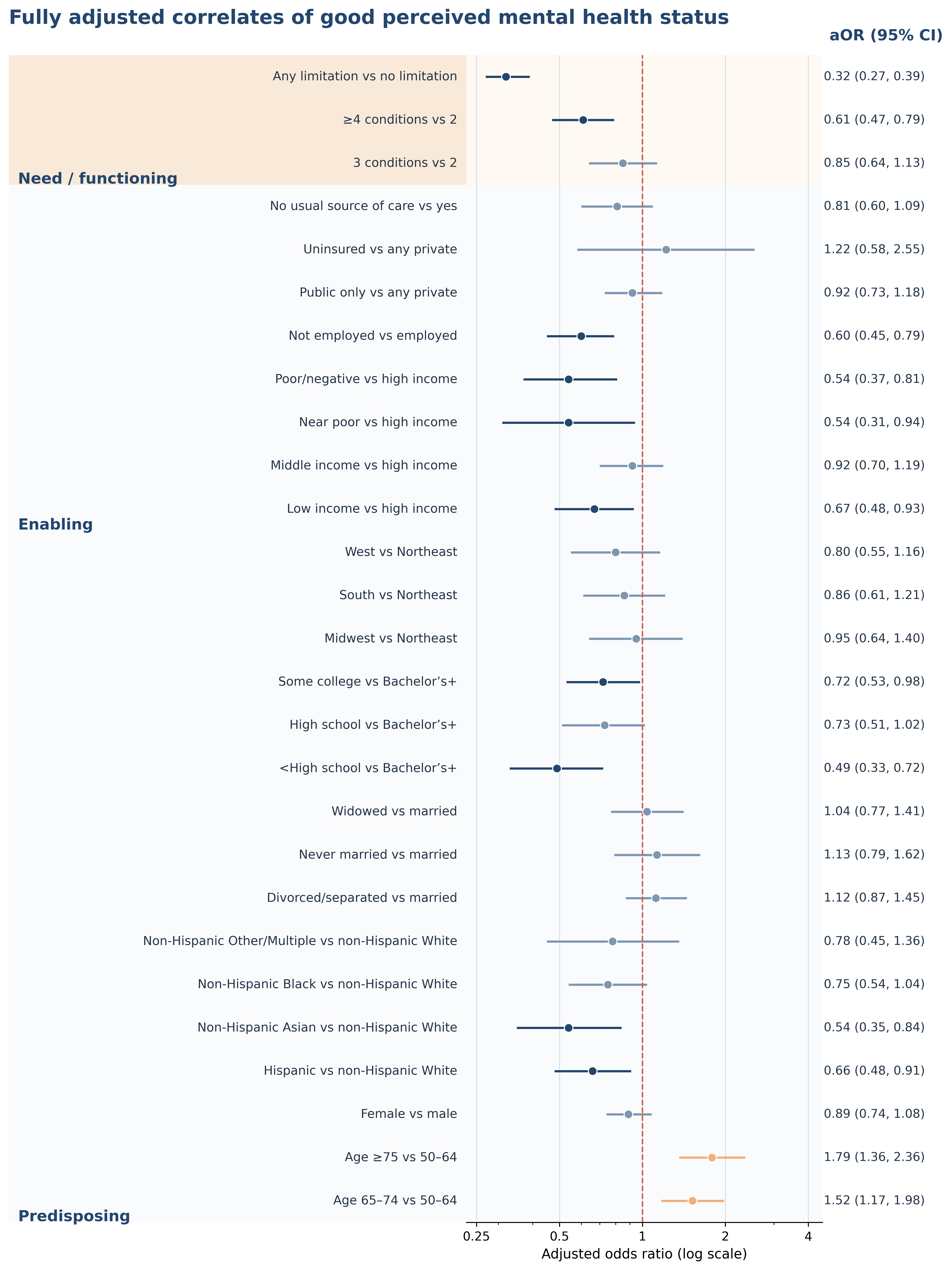

Supplement: Supplementary file 1 [file behavsci-16-00841-s001.zip › Supplementary_File_S2_Figures/Figure_5.png]

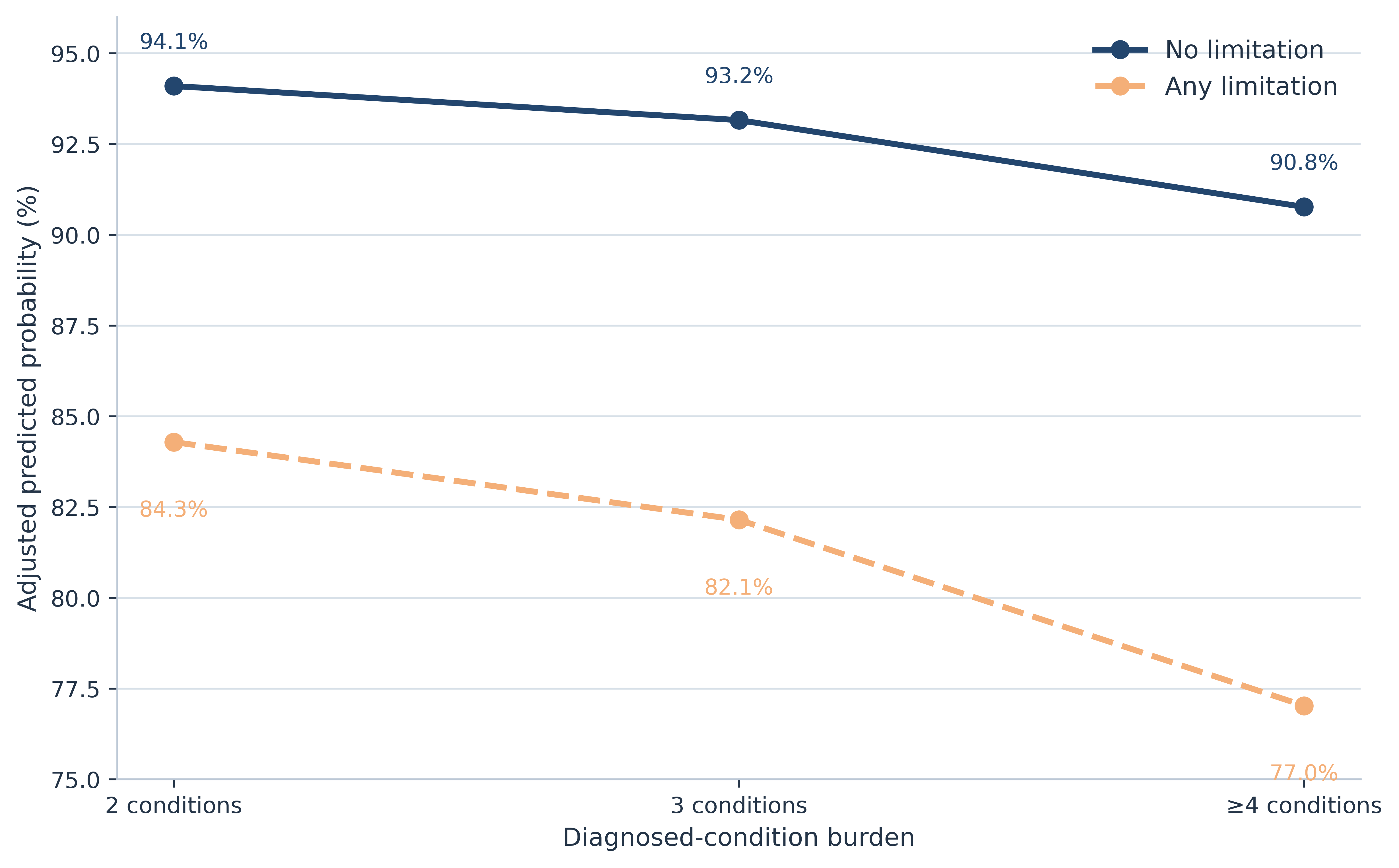

Supplement: Supplementary file 1 [file behavsci-16-00841-s001.zip › Supplementary_File_S2_Figures/Figure_6.png]

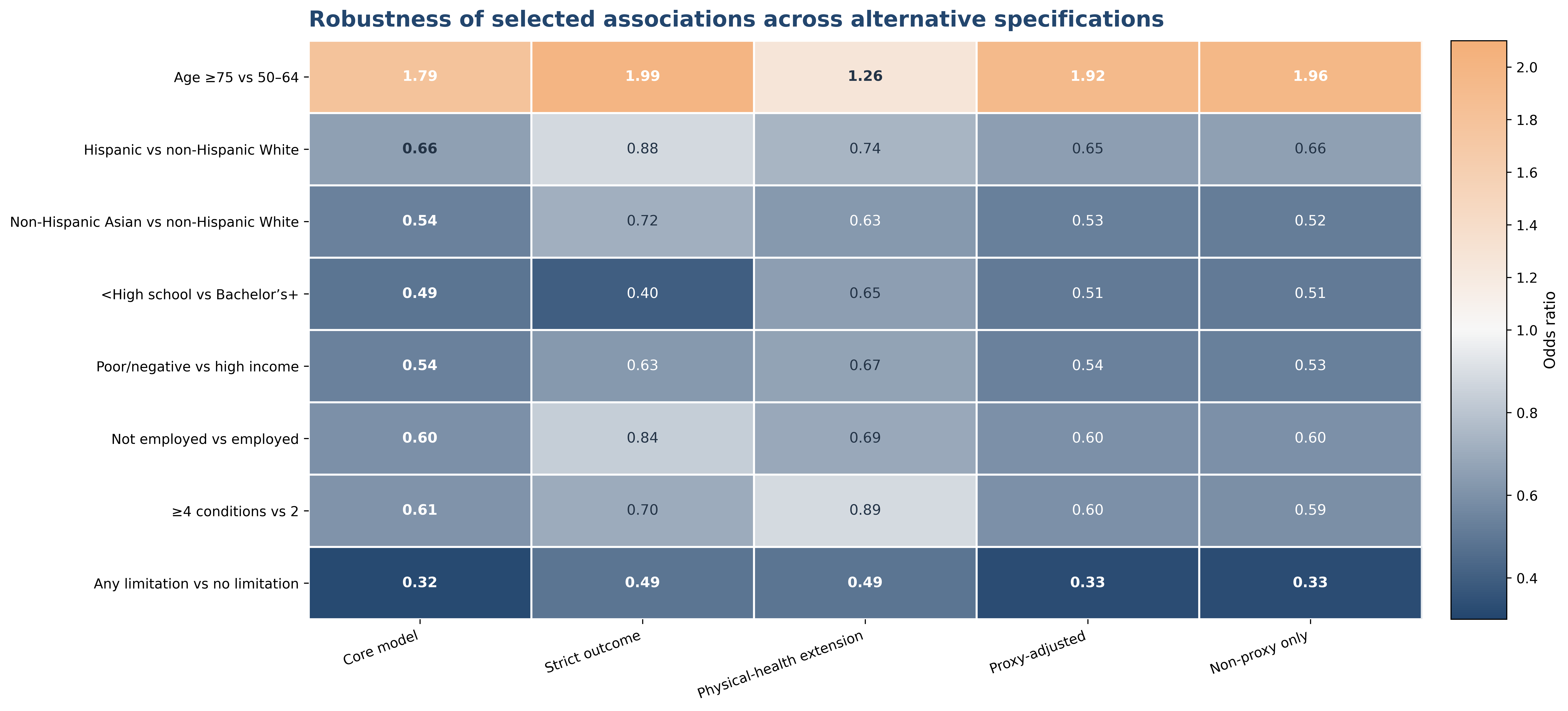

Supplement: Supplementary file 1 [file behavsci-16-00841-s001.zip › Supplementary_File_S2_Figures/Figure_7.png]
